# Case Report: Clinicopathological Analysis of Minute Pulmonary Meningothelial-Like Nodules: Report of 7 Cases

**DOI:** 10.3389/fonc.2022.942517

**Published:** 2022-07-19

**Authors:** Ying-xia Wang, Zi Lei, Man Yang, Zhi-yuan Wang, Xuan Zhang, Guo-qing Pan

**Affiliations:** ^1^ Department of Pathology, The First Affiliated Hospital of Kunming Medical University, Kunming, China; ^2^ Department of Pathology, Fuyuan County People’s Hospital of Qujing City, Fuyuan, China; ^3^ School of Pharmaceutical Sciences and Yunnan Key Laboratory of Pharmacology for Natural Products, Kunming Medical University, Kunming, China

**Keywords:** minute pulmonary meningothelial-like nodules, benign lesions, pathological features, immunophenotype, radiographic manifestations

## Abstract

**Objective:**

To investigate the clinical manifestations, radiologic features, pathological features, and immunophenotype of minute pulmonary meningothelial-like nodules (MPMNs).

**Method:**

This is a retrospective observational study. We collected the clinical data of 7 cases of MPMNs, and performed comprehensive characterization using a combination of clinical, morphological, radiologic and immunohistochemical assessments.

**Results:**

Of the 7 cases of MPMNs, 6 were female and 1 was male. The median age was 55 years. All MPMNs were multiple in lung with the size from 0,01 to 0,5cm. Chest CT examination showed ground-glass attenuation or solid nodules. Four cases were concomitant with carcinoma and/or pneumonia, and 3 cases occurred alone. Four of the 7 patients had no obvious symptoms; 3 patients had chest pain or cough or shortness of breath or hemoptysis. Multiple white nodules were found macroscopically, and the diseased cells grew along the alveolar septum, with relatively normal morphology, rich cytoplasm, unclear cell boundary, and uniform nucleus with delicate chromatin and without atypia; and the diseased cells showed nest or whorls distribution. EMA, PR, CD56 and vimentin were positive in all cases by immunohistochemistry.

**Conclusions:**

MPMNs are rare benign lesions in the lung, often multiple, usually less than 0.5cm in diameter, most of which have no obvious clinical symptoms. MPMNs are often found by chest CT, and occur independently or concomitant with other lesions. The positive immunohistochemical staining of EMA, PR, CD56, vimentin supports the diagnosis.

## Highlights

➢Minute pulmonary meningothelial-like nodules (MPMNs) are rare benign lesions in the lung that most often occur in middle-aged and elderly women.
➢Chest CT examination shows ground-glass attenuation or solid nodules.
➢The morphology of diseased cells shows characteristics of meningothelial cells.
➢Positive immunohistochemistry for EMA, PR, CD56, and vimentin supports the diagnosis of MPMNs.

## Introduction

Minute pulmonary meningothelial-like nodules (MPMNs) are rare, small benign lesions in the lungs that are usually found incidentally in surgical specimens and in routine pathology examinations of autopsy specimens (1, 2). Korn et al. first reported this disease in 1960 (3). The occurrence of MPMNs is associated with many diseases, such as pulmonary thromboembolism, interstitial lung disease, and lung adenocarcinoma; they are sometimes found as concomitant diseases of the main disease of the lung (prevalent in lung cancer cases) or can occur alone (4–6).

MPMNs are usually asymptomatic and are not easily detected clinically. The early diagnosis rate of MPMNs has improved with the popularization of chest thin-section computed tomography (CT) in lung cancer screening (7). The CT presentation of MPMNs is multiple micronodules, usually less than 0.5 cm in diameter, with ground glass-like changes (8). It is difficult to distinguish MPMNs from lung carcinoma *in situ* or microinvasive adenocarcinoma on imaging; thus, intraoperative or postoperative pathological examination and immunophenotypic identification are required to confirm the diagnosis. MPMNs are reactive proliferative lesions of pulmonary meningeal epithelial cells, and their morphologic structure and immunohistochemical examination show that they are similar to meningiomas, with the morphologic features and immunotype of meningeal epithelial cells (9).

As a rare disease, MPMNs have been reported in relatively few studies, with a few reports in out of China and rare reports in China. Therefore, MPMNs are poorly understood by clinicians, radiologists, and pathologists. In this paper, we report 7 cases of MPMNs and review the relevant literature to discuss the clinical manifestations, imaging features, pathological characteristics, and immunophenotypes of MPMNs and summarize their diagnostic points in order to improve physicians’ understanding of this disease.

## Materials and methods

### General Information

The clinical data of 7 patients with MPMNs confirmed by the pathology department of our hospital from December 2020 to April 2021 were collected, among which 3 cases occurred in the lower lobe of the left lung, 3 cases occurred in the lower lobe of the right lung, and 1 case occurred in the upper lobe of the left lung. Multiple nodules with ground glass density were found by CT through Artificial Intelligence(AI) scanning and confirmation of experienced radiologists, and couldn’t be differentiated with carcinorma. The nodules were removed by thoracoscopic surgery for frozen section diagnosis to determine the scope of surgery, and postoperative paraffin sections and immunohistochemical staining were performed for further typing.

### Intraoperative Frozen Pathological Examination

The tissues collected by thoracoscopy were immediately cut to look for the nodules. The lesioned nodules were cut into 1.5x1.5x0.2-cm tissue blocks, treated with a frozen embedding agent, placed in a -20°C frozen sectioning machine. After embedding, the tissues were cut into 5-μm slices, fixed in alcoholic ether for 1 min, and then subjected to hematoxylin and eosin (HE) staining.

### Paraffin Pathological Section Examination

After the frozen sections were cut, the remaining tissue blocks were first fixed in neutral formaldehyde for 6-8 h, put into a dehydrator for overnight dehydration, taken out and embedded into wax blocks using an embedding machine, then cut into 4-μm thin slices. The slices were baked for 30 min, then dewaxed, de-benzened, washed, stained with hematoxylin, restained with blue, stained with eosin, washed, dehydrated, made transparent, and sealed, and their morphological characteristics were observed under optical microscopy.

### Immunohistochemical Staining

The En-Vision 2-step method was used: they were repaired under high pressure, primary and secondary antibodies were added, submitted to color development with diaminobenzidine (DAB), and submitted to hematoxylin staining of cell nuclei, and then underwent dehydration, transparency, and sealing.

## Results

### Clinical and Imaging Features

**Figure 1:**
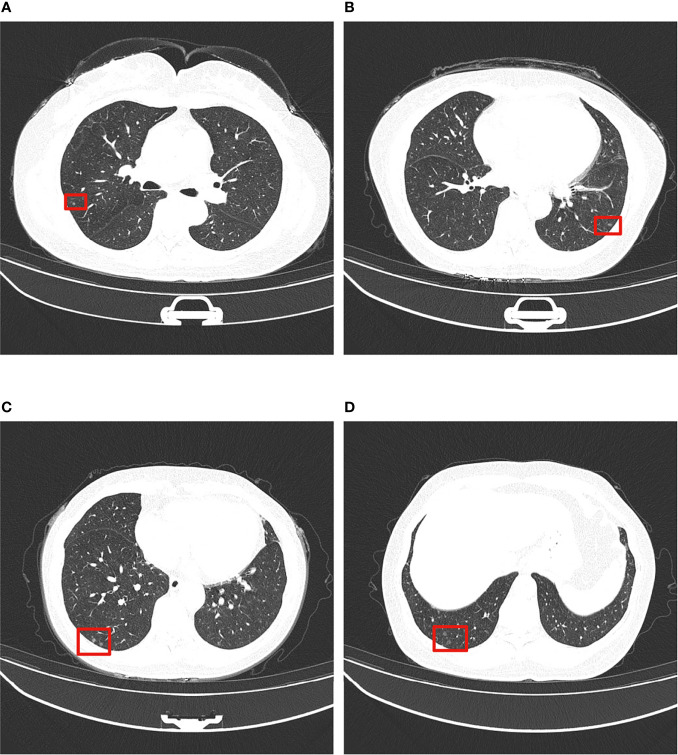
Typical thin-section CT presentation of MPMNs. Multiple ground glass density shadows in the middle and lower lobes of the left and right lungs (shown in red boxes), 0.2-0.4 cm in diameter, with irregular shadows and slightly blurred boundary. **(A–D)** show ground glass density nodules in left and right lungs at different CT sections.

The clinical and imaging data of the 7 cases of MPMNs in this study are shown in **Table 1**. Age-sex distribution: 6 patients were female (31-56 years old, mean 49.5 years old) and 1 case was an elderly male. Number, diameter and location of nodules: all had multiple microscopic nodules <0.5 mm in diameter; 6 patients had nodules in the lower lobe of the lung, and 1 had nodules in the upper lobe. Concomitant lesions: 3 patients had no concomitant lesions, 2 patients had invasive adenocarcinoma of the lung (one of them also had a bronchial adenoma), 1 had nonkeratinizing squamous cell carcinoma and organizing pneumonia, and 1 had organizing pneumonia and atypical adenomatous nodules. Clinical symptoms: 4 patients had no clinical symptoms, and the pulmonary nodules were found during physical examination; 1 patient had chest pain with radiating pain in the right shoulder; 1 patient had cough with a small amount of yellow sputum; and 1 patient had recurrent cough and shortness of breath with hemoptysis. Imaging manifestations: multiple micronodules, mostly ground glass density nodules, were seen on chest CT (**Figure 1**).

**Table 1:** Clinical and Imaging data of 7 MPMN Cases.

Case number	Age (years)	Sex	Location	Number and diameter of nodules	Associated lesions	Symptoms	Radiographic manifestations
1	30-35	Female	Lower lobe of the left lung	Multiple, 0.1 cm	Microinvasive adenocarcinoma of the lung, bronchial adenoma	Asymptomatic; pulmonary nodules found on physical examination	Multiple ground glass nodules with slightly blurred borders
2	40-45	Female	Upper lobe of the left lung	Multiple, 0.1 cm	Multiple invasive lung adenocarcinoma	Asymptomatic; pulmonary nodules found on physical examination	Multiple masses in the left lung and multiple microscopic nodules in both lungs
3	50-55	Female	Lower lobe of the right lung	Multiple, 0.3-0.5 cm in diameter	None	Asymptomatic; pulmonary nodules found on physical examination	Multiple ground glass density micro-nodules in both lungs
4	50-55	Female	Lower lobe of the left lung	Multiple, 0.01-0.05 cm in diameter	None	Asymptomatic; pulmonary nodules found on physical examination	Multiple microscopic nodules in both lungs, some of which were ground glass nodules
5	50-55	Female	Lower lobe of the right lung	Multiple, 0.4-0.5 cm in diameter	None	Chest pain radiating to the right shoulder	Multiple microscopic nodules in both lungs
6	56-60	Female	Lower lobe of the right lung	Multiple, 0.1-0.4 cm in diameter	Organizing pneumonia, atypical adenomatous nodules	Cough with a small amount of yellow sputum	Nodular foci in the dorsal segment of the right lower lobe with multiple micronodules in both lungs
7	66-70	Male	Lower lobe of the left lung	Multiple, 0.1 cm in diameter	Nonkeratinizing squamous cell carcinoma, organizing pneumonia	Recurrent cough, shortness of breath, hemoptysis	Mass in the hilar region of the lower lobe of the left lung with multiple small nodules in both lungs

### Pathomorphological Features

#### Macroscopic Features

Multiple grayish-white solid nodules with medium hardness, irregular morphology and well demarcated borders, 0.1-0.5 cm in diameter, were seen in the specimens of all 7 cases.

#### Intraoperative Frozen Section Microscopic Features

**Figure 2:**
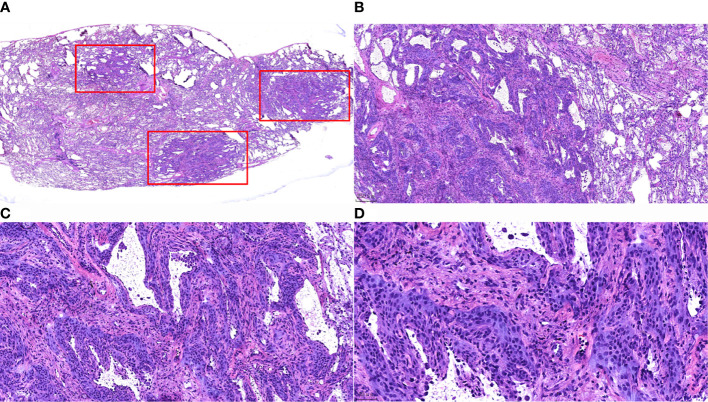
Morphological characteristics of intraoperative frozen sections of MPMNs. **(A)** Frozen section HE staining x7.2. Three nodules (shown in the red box) with irregular morphology and clear borders are visible in the lung tissue. **(B)** x40 shows 1 of the nodules growing around the blood vessels. **(C)** Frozen section, HE staining, x100. The lesion cells are growing along the alveolar septum, with an open air cavity and the proliferation of interstitial fibrous tissue. **(D)** Frozen section, HE staining, x200. Well-differentiated cells were observed without atypia, with a smooth nuclear membrane, a low nucleo-cytoplasmic ratio and no pathological nuclear division.

Widened alveolar septum, visible hyperplastic epithelioid cells growing along the alveolar septum with round or ovoid cells that were uniform in size, arranged in a complex layer, and without heterogeneity. It was difficult to distinguish from atypical adenomatous hyperplasia in frozen sections. Only 1 case was diagnosed as a benign lesion, and the typing was pending postoperative paraffin section results, while the other 6 cases were all diagnosed as atypical alveolar epithelial hyperplasia pending postoperative paraffin section results (**Figure 2**).

#### Postoperative Paraffin Section Microscopic Features

**Figure 3:**
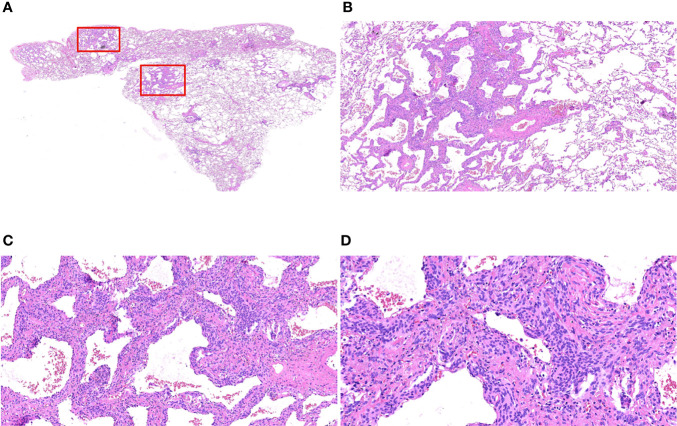
Morphological characteristics of postoperative paraffin sections of MPMNs. **(A)** Paraffin section HE x7.2 with an open alveolar cavity, uniform distribution of fine bronchi, several areas of widened alveolar septa and dense cells (shown in the red box) still clearly demarcated from the surrounding lung tissue, without an envelope. **(B)** x40 HE shows widened alveolar septa, lesion cells growing along the alveolar septa and an open air cavity. **(C)** HE x100 lesion cells partially growing around the blood vessels. **(D)** HE x200 shows that the lesion cells are round or ovoid, with relatively normal morphology, abundant cytoplasm, unclear cell demarcation, light stained nuclei with delicate chromatin and without atypia,.

Microscopically, the lesion was still clearly demarcated from the surrounding lung tissue, without a capsule; the alveolar septa were widened, the lesion cells grew along the alveolar septum, the air cavity was open, and some of the lesion cells grew around blood vessels. The lesion cells were round or ovoid and uniform in size. The cell morphology was relatively normal, without atypia. The cytoplasm was abundant, and the cell boundary was unclear. The nucleus was light stained, and delicate chromatin was evenly distributed (**Figure 3**).

### Immunophenotypic Characteristics

**Figure 4:**
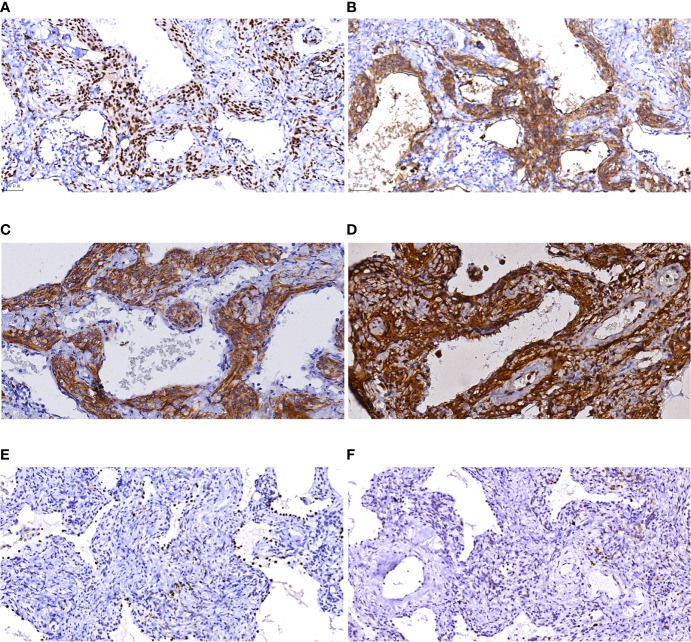
Immunohistochemical results of MPMNs. **(A)** x200 The cells were nuclear positive for PR; **(B)** x200 The cells were cytoplasmic positive for EMA; **(C)** x200 The cells were cytoplasmic positive for CD56; **(D)** x200 The cells were both nuclear and cytoplasmic positive for vimentin; **(E)** x200 Normal type II alveolar epithelial cells were positive and meningeal epithelial-like cells were negative for TTF-1; **(F)** x200 The number of Ki-67 positive cells was <1%.

Of the 7 MPMNs, 7 were positive for PR, EMA, CD56 and vimentin, with 100% positivity; and 7 were negative for Syn, Chromogranin-A, TTF-1, NapsinA, Pan-CK, and CD68, with 100% negativity; the Ki-67 index was < 3% in all cases, with the range from less than 1% to less than 3%, as shown in **Figure 4**.

## Discussion

MPMNs are benign meningeal epithelial-like proliferative lesions occurring in the interstitial lung stroma and are most common in women over 60 years of age; they are often accompanied by other diseases, including lung cancer, chronic lung disease, congestive heart failure, and thromboembolic disease (1, 2, 10–12). Among the 7 patients with MPMNs in this study, there were 6 female patients with an average age of 49.5 and 1 elderly male patient, indicating that MPMNs tend to occur in women and the age of onset tends to be younger, which may be due to the increasing use of chest CT lung cancer screening in routine physical examinations, which has increased the early diagnosis rate of MPMNs. Among the 7 MPMN patients, 3 had no concomitant lesions, 2 had concomitant invasive lung adenocarcinoma (one with accompanying bronchial adenoma), 1 also had nonkeratinizing squamous cell carcinoma and organizing pneumonia, and 1 also had organizing pneumonia and atypical adenomatous nodules. These results suggested that MPMNs can occur either alone or concomitant with other lesions, including malignant or benign tumors and inflammatory lesions.

Patients with MPMNs usually have no obvious clinical symptoms, and the nodules are only detected on chest CT examination or accidentally at visits for symptoms associated with other lung diseases. In the present study, multiple pulmonary nodules were found in specimens of all 7 patients on chest CT examination through Artificial Intelligence(AI) scanning and confirmation of experienced radiologists. Of the 7 patients with MPMNs, 4 had no obvious clinical symptoms, 1 had chest pain radiating to the right shoulder; 1 had cough with a small amount of yellow sputum; and 1 case had recurrent cough with shortness of breath and hemoptysis. The true incidence of MPMNs may be underestimated due to the insidious nature of their clinical symptoms. Clinicians, radiologists, and pathologists should increase their awareness of and concern about MPMNs.

The typical CT presentation of MPMNs is multiple microscopic nodules ranging from 0.2-0.5 cm in diameter with ground glass-like changes (8, 13); a small number of MPMNs also present with diffuse thin-walled cystic cavities on CT (14, 15). All 7 patients with MPMNs in this study presented with multiple microscopic nodules visible on chest CT with diameters in the range from 0.01-0.5 cm; most of the nodules were ground glass density nodules, and no diffuse thin-walled cystic cavities were found, consistent with the typical CT presentation of MPMNs. However, the CT presentation of MPMNs is similar to that of malignant pulmonary nodules, and it is difficult to distinguish MPMNs from malignant pulmonary nodules based only on the imaging presentation, making it prone to misdiagnosis (16). Therefore, the diagnosis of MPMNs needs to be confirmed by pathomorphological examination of surgical biopsy tissue.

MPMNs need to be differentiated from meningiomas, bronchial adenomas and adenocarcinomas *in situ* in terms of pathomorphology. MPMNs are typically characterized by proliferative lung mesenchymal cells with clear borders, usually without an envelope, and homogeneous round or ovoid swirling arrangements of cells that can grow along the alveolar septa or around blood vessels; these cells are without obvious atypia and resemble meningeal epithelial cells, with fine chromatin, inconspicuous nucleoli, and rare mitotic figures (1, 13, 14, 16–18). Meningiomas usually form encapsulated masses with solid, nested clusters of tumor cells, usually without alveolar lumen and residual alveolar epithelial cells. Bronchial adenomas are bilayered structures formed by epithelial cells and continuously arranged basal cells: the epithelial layer is structurally diverse and can be papillary or glandular luminal cells; the cell morphology may be consistent with mucous cells, alveolar epithelial cells, or ciliated columnar epithelial cells without atypia, and exfoliated tumor cells may float in the mucus in the alveolar lumen. Adenocarcinoma *in situ* is usually a monolayer arrangement with no basal cells, atypia of the cells, variable sizes of nuclei, and visible mitotic figures.

In the present study, multiple grayish-white solid nodules with medium hardness, irregular morphology and well demarcated borders, 0.1-0.5 cm in diameter, were found macroscopically in the specimens of all 7 cases. Microscopically, the lesion was still clearly demarcated from the surrounding lung tissue, without an capsule; the alveolar septa were widened, the lesion cells grew along the alveolar septum, the air cavity was open, and some of the lesion cells grew around blood vessels. The lesion cells were round or ovoid and uniform in size. The cell morphology was relatively normal, without atypia. The cytoplasm was abundant, and the cell boundary was unclear. The nucleus was light stained, and delicate chromatin was evenly distributed. All pathological findings mentioned above were consistent with the typical pathological morphological features of MPMNs.

Immunohistochemical detection of specific cellular markers can clarify the cellular immunophenotypic characteristics and determine the cellular origin. In this study, the immunohistochemical results revealed that PR, EMA, CD56, and vimentin were all positively expressed and Chromogranin-A, TTF-1, Napsin A, and CD68 were negative in 7 cases of MPMNs; additionally, the Ki-67 index was <3%, suggesting that the proliferating cells were consistent with the phenotypic characteristics of meningeal epithelial cells, which is consistent with literature reports (7, 16, 19). As a result of the combination of microscopic pathomorphological features and immunophenotypic characteristics, the diagnosis of MPMNs was confirmed in all 7 cases.

All 7 patients of this study were followed up for 13 to 17 months. All patients received chest CT examination once every six months during follow-up period and no nodules were found. Six patients recovered well without symptoms or adverse events after removal of nodules by thoracoscopic surgery, suggesting that the prognosis of MPMNs is good. Pleural effusion and abdominal pain occurred in only 1 patient combined with multiple invasive lung adenocarcinoma, however, both chest CT and abdominal ultrasonography showed no nodules or mass.

## Conclusion

In summary, minute pulmonary meningothelial-like nodules(MPMNs) are rare benign lesions in the lung that most often occur in middle-aged and elderly women. They are often multiple, usually less than 0.5 cm in diameter, most often do not have obvious clinical symptoms, are often detected by chest CT, and can occur alone or concomitant with other lung lesions. Positive immunohistochemistry for EMA, PR, CD56, and vimentin supports the diagnosis. Clinicians, radiologists, and pathologists should increase their attention to and awareness of this disease. Our report will provide references for diagnosis of MPMNs.

## Strengths and limitations of this study

This is a rare case series, MPMNs have been reported in relatively few studies, with a few reports out of China and rare reports in China.

This study performed comprehensive characterization of MPMNs using a combination of clinical, morphological, radiologic and immunohistochemical assessments.

There were only 7 cases in this study and the results were not checked using statistical tools.

## Data Availability Statement

The original contributions presented in the study are included in the article. Further inquiries can be directed to the corresponding authors.

## Ethics Statement

The studies involving human participants were reviewed and approved by Ethical Review Board of the First Affiliated Hospital of Kunming Medical University. The patients/participants provided their written informed consent to participate in this study. Written informed consent was obtained from the participant for the publication of this case report.

## Author Contributions

Y-xW, ZL and MY have contributed equally to this work and share first authorship. Y-xW collected data, wrote the manuscript. ZL designed the project, analyzed the data and revised the paper. MY Collected and analyzed the data. XZ and G-qP designed the project and revised the paper. Z-yW assisted in case collection and analyzed the data. All authors contributed to the article and approved the submitted version.

## Funding

This study was supported by grants from the National Natural Science Foundation of China (No. 81660431), the Joint Projects of Applied Basic Research of Kunming Medical University and Yunnan Provincial Department of science and Technology(No. 2019FE001-220), the Science Project of Yunnan Provincial Department of Education (No. 2018JS202).

## Conflict of Interest

The authors declare that the research was conducted in the absence of any commercial or financial relationships that could be construed as a potential conflict of interest.

## Publisher’s Note

All claims expressed in this article are solely those of the authors and do not necessarily represent those of their affiliated organizations, or those of the publisher, the editors and the reviewers. Any product that may be evaluated in this article, or claim that may be made by its manufacturer, is not guaranteed or endorsed by the publisher.
